# EnBloc Resection of a Chordoma of the Thoracic Spine by “L”-Shaped Osteotomy for Spinal Canal Preservation

**DOI:** 10.3390/jcm14020349

**Published:** 2025-01-08

**Authors:** Alessandro Gasbarrini, Stefano Pasini, Zhaozong Fu, Riccardo Ghermandi, Valerio Pipola, Mauro Gargiulo, Marco Innocenti, Stefano Boriani

**Affiliations:** 1 Spine Surgery Department, IRCCS Istituto Ortopedico Rizzoli, 40136 Bologna, Italy; alessandro.gasbarrini@ior.it (A.G.); riccardo.ghermandi@ior.it (R.G.); valerio.pipola@ior.it (V.P.); 2 Department of Biomedical and Neuromotor Sciences, Alma Mater Studiorum University of Bologna, 40126 Bologna, Italy; 3 Department of Spine Surgery, Jiangmen Central Hospital, Jiangmen 529030, China; fzhz@hotmail.com; 4 Vascular Surgery Department, IRCCS Policlinico di Sant’Orsola, 40138 Bologna, Italy; mauro.gargiulo2@unibo.it; 5 Orthoplasty Department, IRCCS Istituto Ortopedico Rizzoli, 40136 Bologna, Italy; marco.innocenti@ior.it; 6 Alma Mater Studiorum University of Bologna, 40126 Bologna, Italy; stefano.boriani@unibo.it

**Keywords:** enbloc, chordoma, primary bones tumor, spine, tumor resection, new technique, navigation, robotic surgery

## Abstract

**Background/Objectives**: EnBloc resections of bone tumors of the spine are very demanding as the target to achieve a tumor-free margin specimen (sometimes impossible due to the extracompartimental tumor extension) is sometimes conflicting with the integrity of neurological functions and spine stability. **Methods**: The surgical treatment of a huge multi-level chordoma of the thoracic spine with unusual extension is reported. Anteriorly, the tumor widely invaded the mediastinum and displaced the aorta; on the left side, it expanded in the subpleuric region; posteriorly, it was uncommonly distant 13 mm from the posterior wall. **Results**: EnBloc resection is largely performed for primary bone tumors of the spine and many reports have been published concerning brilliant solutions to difficult issues of surgical anatomy. One of the major challenges is still the compatibility between oncological and functional requirements. **Conclusions**: Oncological staging, careful imaging analysis, a multidisciplinary surgical team, and utilization of the most recent technologies like navigation and robotics have made an oncologically appropriate EnBloc resection of a multi-level chordoma of the thoracic spine possible without affecting the continuity of the spinal canal and without any involvement of its content by an original “L”-shaped osteotomy.

## 1. Introduction

The surgical techniques known as *enbloc resections* were designed to resect bone tumors of the limb to replace amputations and are globally known as “*limb salvage surgery*” [1,2]. The possibility to spare the functions of a limb is allowed—in high-grade malignancies—by the local effect of neo-adjuvant chemotherapy (reduction in tumor volume, better definition of the margins) [3].

The same techniques were extended to low-grade malignancies and to aggressive benign tumors, not sensitive to chemotherapy, reducing the local recurrence rate [4]. The local and systemic outcome was correlated to the pathological evidence of the integrity and the thickness of the healthy tissue surrounding the tumor, the so called “*margin*” [1].

Bertil Stener became the pioneer of performing *enbloc* resection in the spine by scrupulously describing the planning of each surgical procedure tailored to the tumor location and extension in order to achieve a tumor-free margin resection [5]. Roy Camille [6], and later Tomita [7], popularized techniques of *spondylectomy* unrelated to the tumor extension and less respectful of the oncological validity of *margins.* As consequence of the spread of commitment to oncological principles, many surgeons started to propose—in the spine also—strategies to adapt the surgical technique to the tumor location and extension, with the primary target of achieving an appropriate *margin* [8,9,10,11,12,13]. Unfortunately, in many cases, this cannot be achieved without functional sacrifices possibly not accepted by the patient [14,15,16] or without including highly morbid steps, like resection of the involved aorta inside the tumoral mass and bypass [17,18]. In recent years, the literature on *enbloc resection* in the spine has been widely populated, with single reports of demanding cases and tips on techniques [19,20,21,21,22,23,23,24,25,26], some retrospective studies from single centers [27,28,29,30,31,32,33,34,35], and a systematic review [36]. An attempt to categorize the different surgical techniques of enbloc resection according to tumor extension was published by Tomita—the original paper, specifically dedicated to metastases [37]. More recently, based on the criteria that make an oncologically valid resection possible and according to tumor extension and location, a classification of possible strategies, including seven combinations of approaches, was recently proposed [38], making reference to the WBB (Weinstein–Boriani–Biagini) staging system [39]. According to this paradigm, when a tumor in the thoracic and lumbar spine is widely extended in the anterior and antero-lateral region of the spine (layer A according to WBB), an anterior approach is preconized first to grant the appropriate margin and a posterior approach second to release the content of the spinal canal and finalize the tumor removal. This strategy is staged as type 3. The tumor extension of a recently observed case of T9 to T11 chordoma was unusual: imaging studies showed a huge extension in the mediastinum but without involvement of the posterior part of the vertebral body. A type 3 resection strategy—not considered in the previously cited article [38]—was planned from the perspective of performing a resection without opening the spine canal (reducing the risks of neurological damage and of spine instability) and without violating the tumor margin.

## 2. Case Report

### 2.1. Clinical Findings

An adult female aged 56 was referred, complaining of lumbar pain for six months. Once a huge tumoral lesion was found arising from T9, a trocar CT scan-guided biopsy was performed through the left pedicle, which allowed for the diagnosis of chordoma.

At admission, the general conditions were excellent, and back pain was reported as being located in the thoracic area, with circumferential irradiation to the sternum. The pain was reported as interfering with sleep and was slightly reduced during daily activities, and no peripheral pain was reported. Opioid pain killers were effective.

### 2.2. Imaging Studies

**Figure 1:**
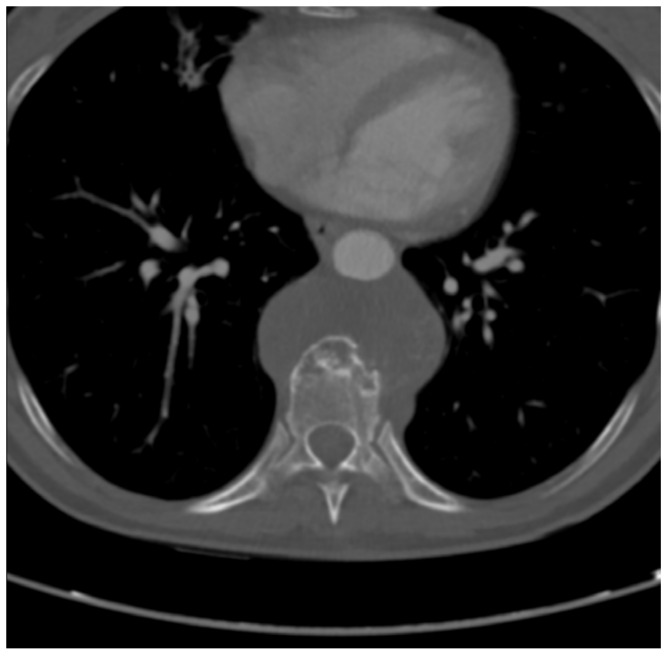
CT scan transverse image crossing T9 shows a lytic, moth-eaten change involving the anterior part of the T9 vertebral body with huge anterior mediastinal soft tissue partially surrounding and displacing the aorta. The transpedicular trocar track for biopsy is visible.

**Figure 2:**
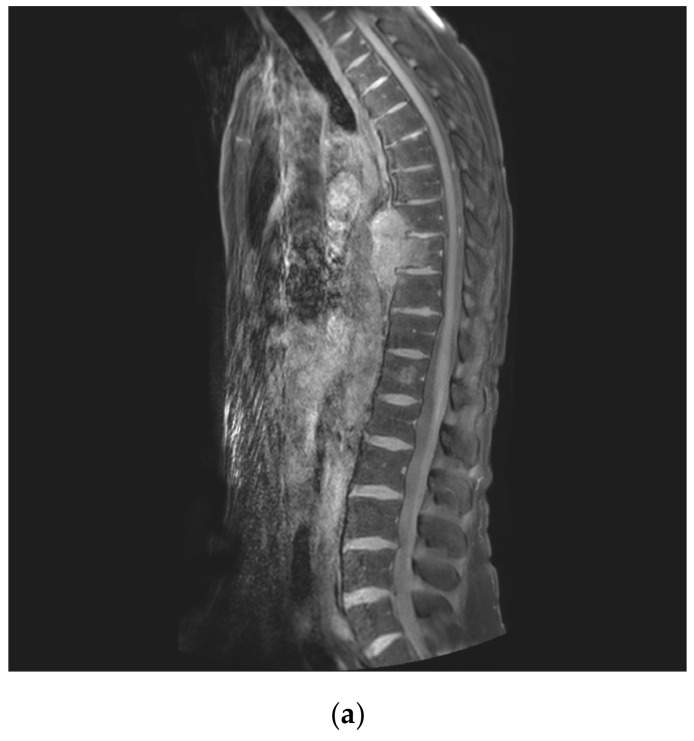

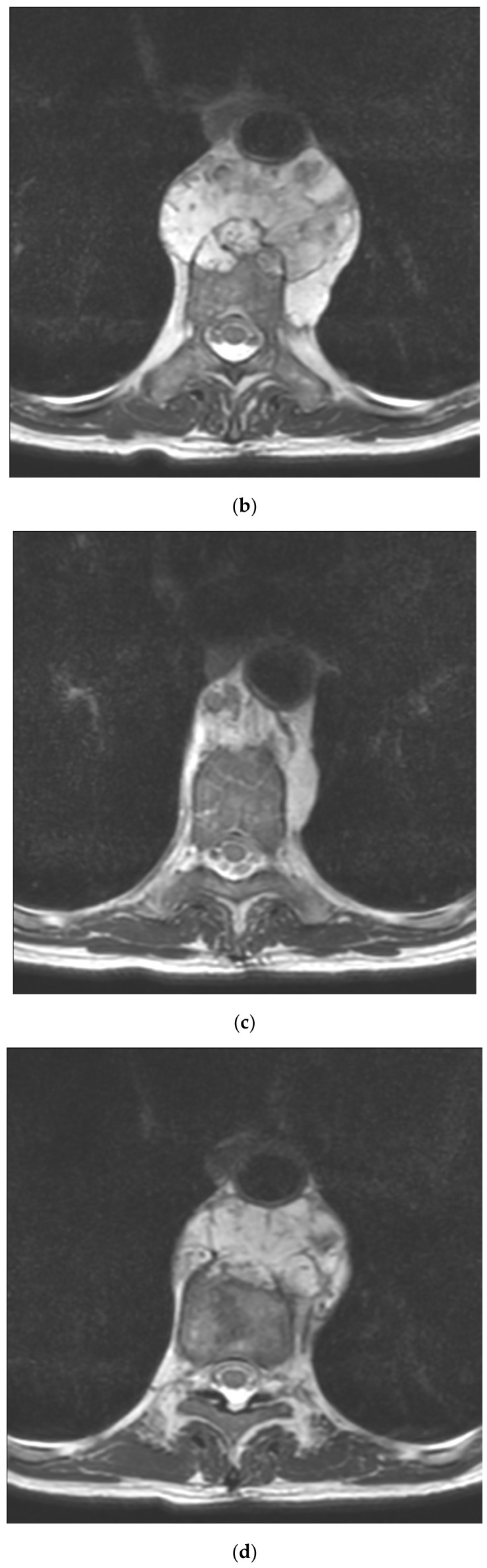
(**a**) MR (T1_mDIXON_TSE) sagittal image shows the longitudinal tumor extension in the mediastinum originating from the vertebral body of T9 and invading the anterior part of T8 and T10 vertebral bodies without involvement of the disks. The disks T7–T8 and T10–T11 seem the best levels for cranial and caudal resection levels. (**b**) MR (T2W_mDixon_TSE FS) transverse image at T9 level clarifies that the aorta wall is not infiltrated and that there is a tumor-free zone from the posterior tumor margin and the posterior vertebral wall (according to the WBB staging system: layer A, only part of layer B, no layer C involvement). At this level (the largest tumor extension), the AP diameter of the T9 vertebral body is 28 mm, and the distance between the tumor and the posterior wall is 13 mm. The tumoral soft tissue expands on left side in the retro-pleural space to the level of the cost-transverse joint in T9 (sectors 8 to 4 according to the WBB staging system). (**c**) MR (T2W_mDixon_TSE FS) transverse image at T8: the tumors is much smaller in the mediastinum end and it is invading only the peripheral vertebral body cortex. The retro-pleural invasion on the left arrives at the level of the posterior longitudinal ligament (sectors 7 to 5 according to the WBB staging system). (**d**) MR (T2W_mDixon_TSE FS) transverse image at T10: the tumor invades the anterior part of the vertebral body; sectors 4 and 5 are not involved in the tumor.

The CT scan shows a lytic, moth-eaten bone lesion in the anterior part of the T9 vertebral body (Figure 1), with huge soft tissue mass better described by MRI as invading the mediastinum and contouring the aorta. The tumor tissue invades the anterior part of the T8 and T10 vertebral bodies without involvement of the disks (Figure 2a). The tumoral soft tissue expands on left side in the retro-pleural space up to the level of the cost-transverse joint in T9 (sector 8 to 4 according to the WBB staging system) (Figure 2b), while in T8 (Figure 2c) and T10 (Figure 2d) sectors 4 and 5 are not involved in the tumor.

A wide tumor-free zone can be seen from the posterior tumor margin and the posterior vertebral wall in the transverse image of all three vertebral bodies (layer A, only part of layer B, no layer C involvement). At the level of the largest tumor extension, the AP diameter of the T9 vertebral body was 28 mm, and the distance between the tumor and the posterior wall was 13 mm (Figure 2b).

### 2.3. Surgical Planning

To achieve tumor-free margins, and considering the tumoral extension, the surgical planning could include the following.

An antero-lateral approach to release the tumor mass from the mediastinal structures, namely from the aorta. A vascular surgeon should be part of the multidisciplinary team and be ready to perform a graft if the aorta must be resected and included in the tumor specimen, in case of involvement of the aortic wall inside the tumor mass.

A posterior approach to complete the circumferential release of the spine without any opening of the spinal canal and to finalize the enbloc resection. An “L”-shaped osteotomy has been designed, with the longest leg in the coronal plane, cutting between the posterior tumor margin and the posterior vertebral wall, and the shorter leg in the sagittal plane, to achieve an appropriate margin to the tumoral extension in the retropleural space facing sector 4. The costo-vertebral joint at T9 will therefore be included in the tumoral specimen.

The osteotomy lines were planned to achieve an appropriate tumor-free margin of the specimen and contemporary to save the continuity of the spinal canal without any involvement of its content. Navigation and robotic guidance are reliable and valuable tools to perform such demanding lines with a precision of few millimeters’ tolerance, as the distance between the tumor and the posterior wall is only 13 mm and the target is to leave at least 5 to 7 mm of healthy bone as a margin over the tumor.

**Figure 3:**
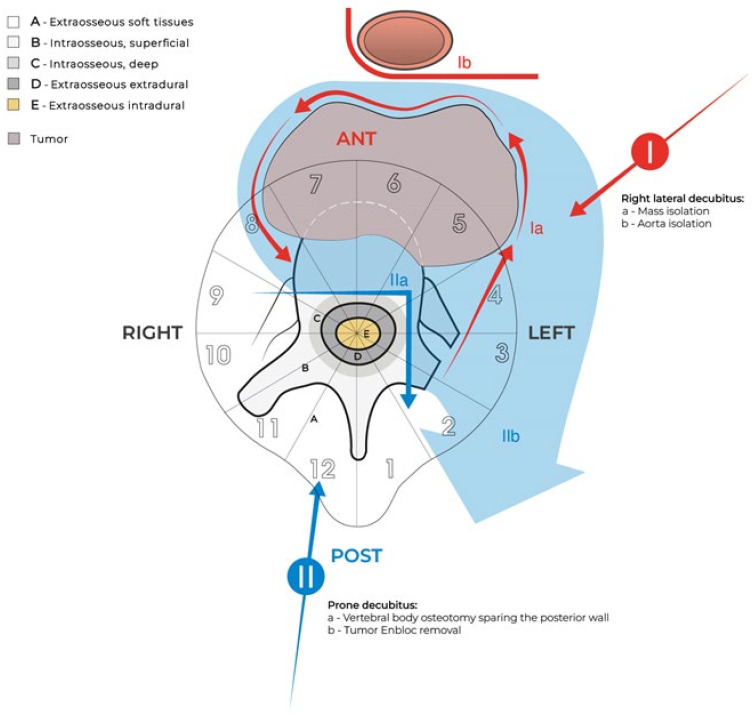
WBB planning of enbloc resection of the reported case. The tumor–free area between the posterior tumor margin and the posterior vertebral wall allows us to consider the possibility of saving the continuity of the spinal canal. Targeting the tumor-free margin and the integrity of the canal surgery should also be performed.

According to WBB-based surgical planning [38], type 1 (single anterior approach) has no possibility of achieving a tumor-free margin specimen due to the eccentric extension into the retropleural space. A second posterior approach with the original “L” osteotomy can be staged as a further sub-type of type 3 [38] (Figure 3).

Pre-operative angiography and selective embolization of the T8, T9, and T10 segmental arteries can be useful to not cause ischemia of the lesion but to embolize those arteries stretched by the tumor growth, which are sometimes difficult to detect. A tear of these stretched arteries may not immediately cause bleeding but can produce dangerous hematomas in the early post-operative course when the arterial walls relax and blood pressure increases.

First step (I): Right lateral decubitus. Antero-lateral left trans-pleural approach to release the tumor mass from mediastinal structures, namely the descending aorta, and partial diskectomies at T7–T8 and T10–T11: a left posterolateral thoracotomy is performed at the level of the sixth intercostal space. Once the pleural cavity is opened, the neoplasm can be appreciated at the level of the T8, T9, and T10 bodies. The pulmonary ligament is dissected, and the parietal pleura is opened, isolating the mass. Finally, partial costectomy of the VII, VIII, IX, and X ribs is performed. Diskectomies complete the first surgical step.

Second step (II): Prone position. The posterior approach includes conventional lateral release of the vertebral bodies—once the posterior segment of three ribs is sacrificed—until the tumor mass is reached. “L-shaped” osteotomy from the T7–T8 to T10–T11 disk level and specimen removal are performed.

### 2.4. Surgical Procedure

**Figure 4:**
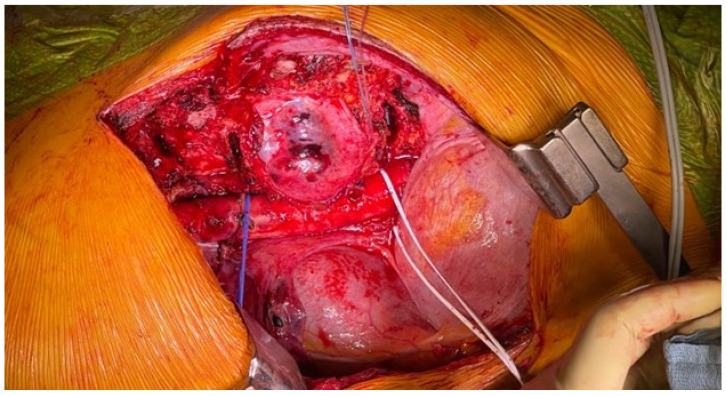
Intraoperative image at the end of the left antero-lateral trans-pleural approach (at the seventh rib). The tumor mass can be seen covered by the parietal pleura as an effective margin. The descending aorta has been isolated and fully released without violations of the tumor mass. The segmental vessels are ligated at the origin. The anterior part of disks T7–T8 and T10–T11 has been excised. The left T7 segmental artery is prepared for planned anastomosis with the fibula feeding artery. Before the closure, a Gore–Tex mesh is positioned over the tumor mass for protection and to easily find the safely released peritumoral area during the posterior approach.

**Figure 5:**
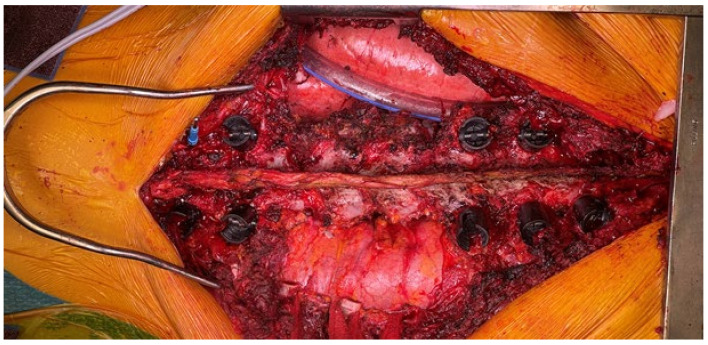
Second stage. Patient in prone position. Posterior midline approach, and positioning of Carbon Fiber-Reinforced PEEK (CFRP) (Carboclear by Carbofix), screws in the pedicles of T5, T6, T7, T11, and T12. Excision of rib segments proximal to the spine at T8, T9, and T10; lateral release of the vertebral bodies reaching Gore–Tex mesh left over the released area during the anterior approach.

**Figure 6:**
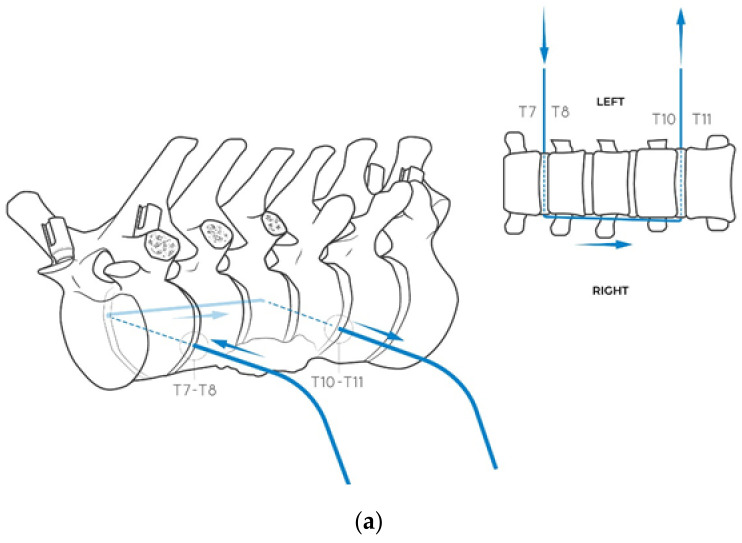

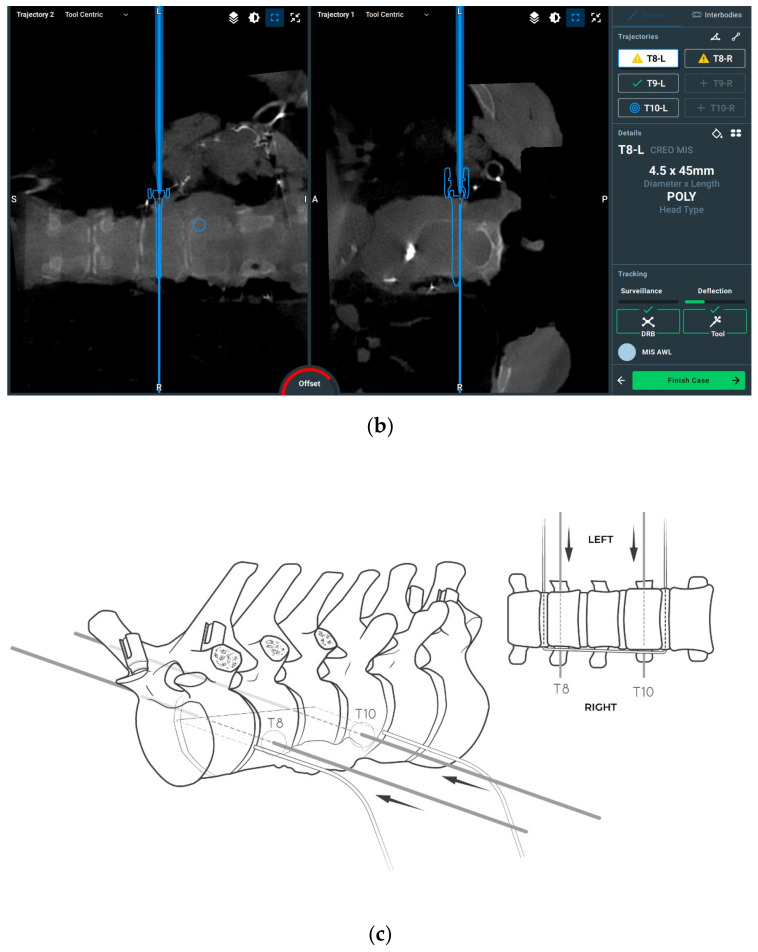
(**a**) The Tomita saw is inserted left to right in the interspace T7–T8 inside the excised disk, leaving the posterior part of the disk intact. Its extremity is shifted along the right side of the spine up to disk T10–T11 (whose posterior half is similarly spared), where it is retrieved to the left side. (**b**) The anesthesiologist is asked to collapse the left lung in order to gain access to the lateral aspect of the vertebral bodies without injuring the parenchyma. By surgical robot (Excelsius by Globus) guidance, two percutaneous K wires are introduced on the coronal plane left to right, just below the superior endplate of T8 and just above the inferior endplate of T10, at a safe distance from the tumor and the vertebral posterior wall. Navigation is necessary in this phase because it is not possible to visualize the tumor inside of the vertebral body, and navigation is only able to guide the insertion, avoiding tumor violation. (**c**) The K–wires are introduced slightly dorsal to the plane of the Tomita saw to guide the osteotomy on the correct coronal plane to save the posterior part of the vertebral bodies without violating the tumor mass (arrows suggest the direction left–to–right).

**Figure 7:**
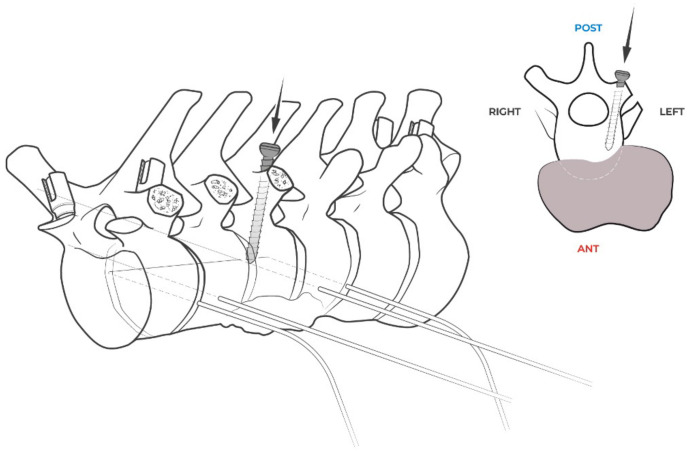
A screw is positioned in the left pedicle of T9 (uninvolved by the tumor) to stop the coronal osteotomy and to act as a pivot to start a sagittal osteotomy ending in “L-shaped” osteotomy. Again, screw insertion is guided by surgical navigation in order to avoid the end of the screw reaching the margin of the tumor.

**Figure 8:**
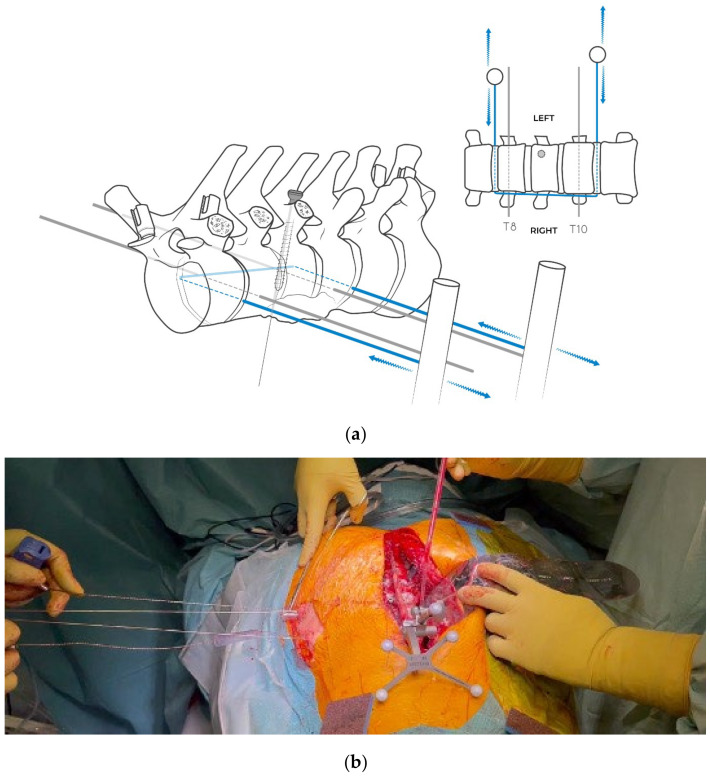
(**a**) The extremities of the Tomita saw are exteriorized by short incisions in order to cut on the desired coronal plan, running on the guide of the couple of K-wires. (**b**) Intraoperative picture of the Tsaw osteotomy on the coronal plane, always under navigation system control. To avoid damage to the soft tissues during the resection on the coronal plane, two plastic cannulas are used, which are positioned through the chest wall and inside which the saw is slid. In the image, while the first operator keeps the saw wire tensioned, the second operator positions the proximal cannula with Kocher pliers.

**Figure 9:**
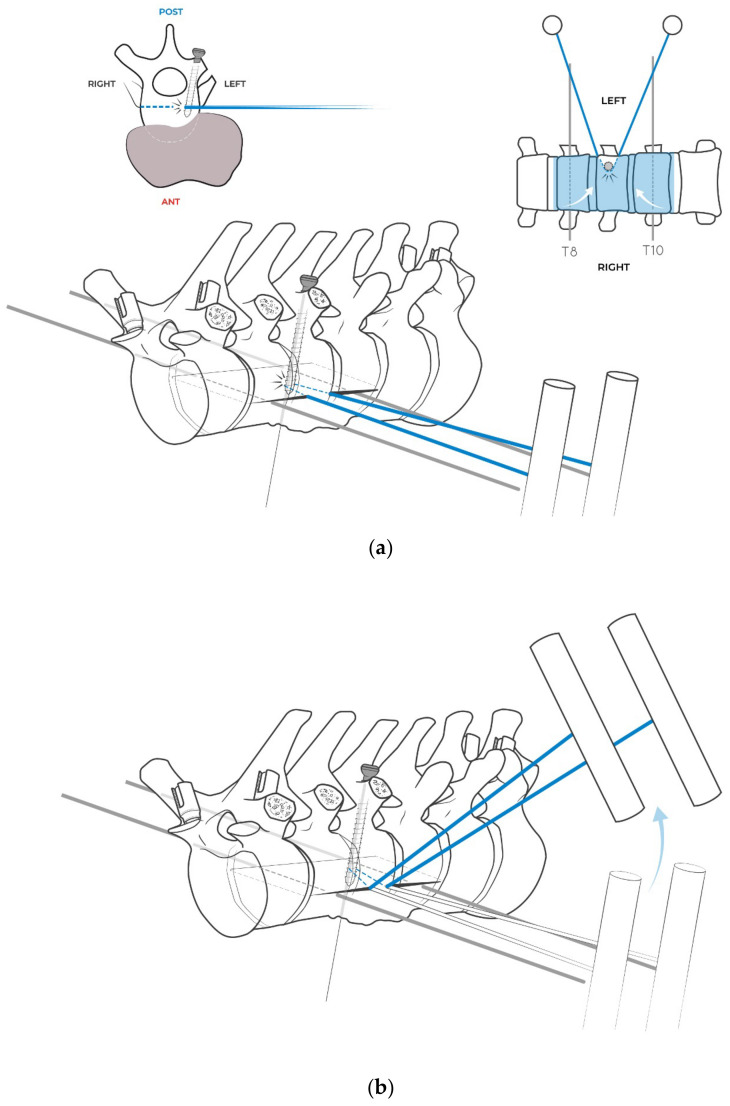

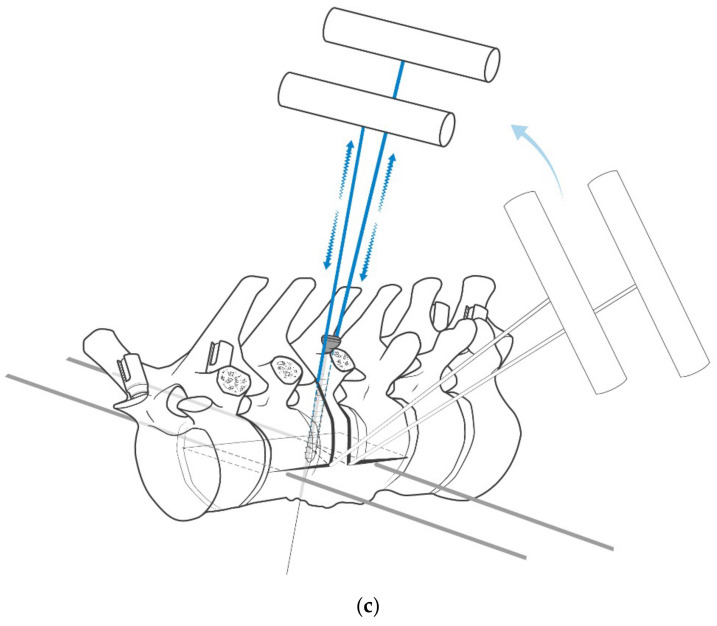
(**a**) As soon as the saw comes into contact with the screw positioned in the left pedicle of T9, the handles of the saw are removed and the two extremities of the wire are slid back through the percutaneous paths. (**b**) The two extremities of the wire are then brought out vertically through the surgical wound. The handles are assembled on the wire again and repositioned along a sagittal direction. (**c**) Pulling up the handles, with the wire oriented in a vertical direction along the sagittal plane, the “L” osteotomy is completed at the T9 level, where the tumor grows laterally to sector 4. The T9 screw is therefore used first as a constraint to stop the saw along the resection in the coronal plane, and then as a guide for the osteotomy on the sagittal plane, providing the correct direction and avoiding a deviation of the saw.

**Figure 10:**
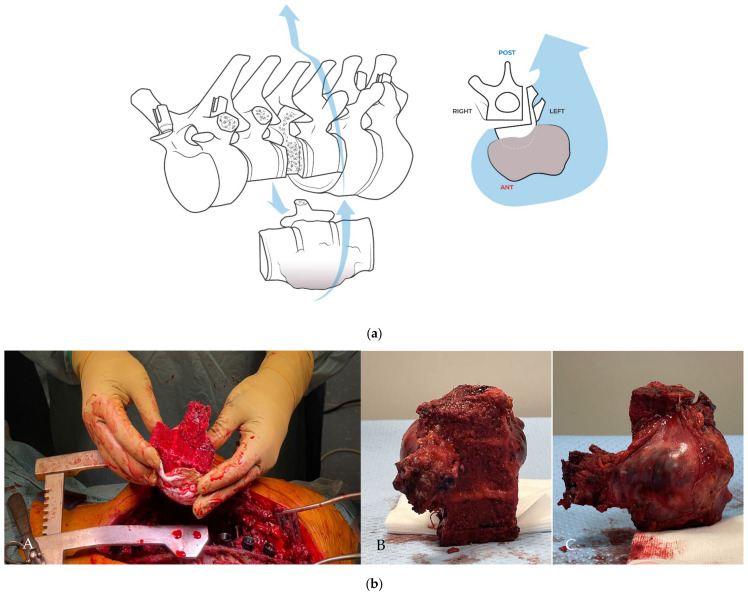
(**a**) By careful manual manipulations, the specimen was removed. (**b**) The tumor, covered by a thick margin of healthy tissue and still protected by the Gore-Tex mesh, was removed from the left side. The coronal resection of the vertebral bodies of T8 and T10 and the effect of the sagittal leg of the “L-shaped” osteotomy can be seen, allowing the inclusion of sectors 4 and 5 of T9 in the enbloc resection, corresponding to the lateral retropleural tumor extension (panel **A**) and to other angles of the excised specimen in panels (**B**,**C**).

**Figure 11:**
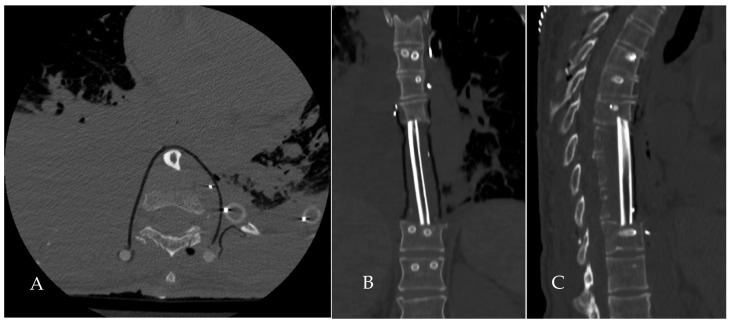
Post-operative CT scan image at the lower T9 level, distal to the sagittal leg of the osteotomy. As planned, the coronal osteotomy spared the circumference of the spinal canal and runs some millimeters from the tumor posterior margin. A Gore-Tex sheet is left, creating a new compartment from the mediastinum. Panel (**A**), axial section. Panels (**B**,**C**), coronal and sagittal sections showing the grafted fibula.

The surgical procedure is illustrated in Figure 4, Figure 5, Figure 6, Figure 7, Figure 8, Figure 9, Figure 10 and Figure 11 and described in the corresponding legends. The distinctive step of this original procedure is the robot-guided coronal cut by a transcutaneous Tomita saw and the conversion to a sagittal cut by pivoting on a pedicle screw. From an oncological point of view, the precision of the robotic–assisted positioning of the saw allows one to achieve a tumor-free margin, and pivoting over the screw allows for resection of the tumor involved in the left posterior part of the vertebral body. From a mechanical point of view, this technique allows one to spare the wall of the spinal canal and the enbloc resection is performed without encroaching on the epidural space.

The original plan was to remove the screw together with the specimen, but during the osteotomy on the sagittal plane, the saw became stuck in the threads of the screw. For this reason, the screw was removed, and the resection was completed free–hand. This was possible because the saw became stuck during the final part of the resection, and when we were therefore already in a safe zone. This problem could be avoided by using a partially threaded screw.

### 2.5. Reconstruction

A sound posterior fixation by a pedicular screw and rods (three levels above and two levels below for a final T5-T12 construct) was performed using a PEEKReinforced Carbon Fiber system (Carboclear by Carbofix). This titanium-free material has the same—if not better—mechanical properties as titanium [40], but it is radiolucent and allows for the early detection of a recurrence and a better radiotherapy planning as delivers a significant reduction in artifacts [41], allowing for a better definition of the target and organs at risk [42]. In case of accelerated particles, radiotherapy is indicated, and the backscattering effect by PRCF implants is significantly reduced, resulting in a higher dose on the tumor and a lower irradiation of neighboring organs [43].

The anterior column loss only concerns the anterior half of bodies and disks, as the coronal osteotomy allowed us to save the posterior vertebral walls. To grant anterior stability, a segment of autologous vascularized fibula was inserted between the upper and lower bone surfaces left by the “L” osteotomy.

The feeding artery of the fibula was anastomized with the previously prepared intercostal artery during the first surgical step.

### 2.6. Pathologist’s Report

**Figure 12:**
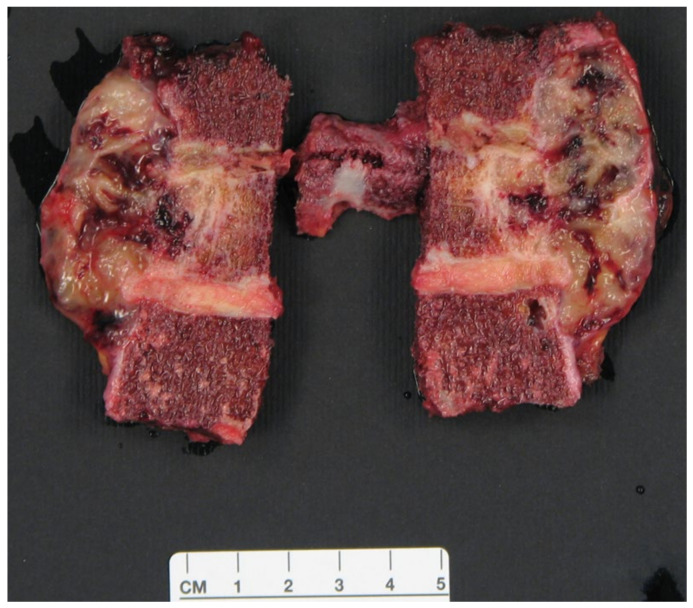
Pathologist’s specimen of the resected vertebrae.

The diagnosis of chordoma was confirmed. The *margin* was defined “marginal” at the anterior part of the tumor, expanding into the mediastinum, represented by a thin layer of capsule and reactive tissue and partially covered by the pleura. The *margin* at the bone was calculated to be 3 to 5 mm, and intact. This was also defined as “marginal” (Figure 12).

### 2.7. Post-Operative Period and Follow Up

The patient was hospitalized for 19 days after surgery and the post-operative period was complicated by a pleural effusion which progressively decreased. After five months, she was re-admitted because of a post-surgical infection due to Staphylococcus Aureus that required a reintervention of debridement associated with long-term antibiotic therapy. Six months after the reintervention, no signs of infection or local recurrence were detected with MRI. The grafted fibula appeared stable with bone integration in progress, as shown by CT scan. The absence of local recurrence was confirmed at the last follow up, 15 months after the primary surgery.

## 3. Discussion

The surgical oncology of bone tumors requires the careful planning of enbloc procedures: the soft tissue dissection and the osteotomy lines must be decided by considering the distance from the tumor periphery, which anatomical structures can be released, and which to be sacrificed for tumor-free margins. The pioneer of enbloc resection who planned to achieve an appropriate margin was Bertil Stener [5]. He planned the surgical steps based on his own designs. Nowadays, high-resolution imaging studies allow surgeons to better understand the details of tumor extension and anatomical element involvement; 3D images and 3D-printed plastic specimens are extremely useful to visualize the surgical anatomy [44]. Newer technologies allow us to improve the accuracy of the resection, develop new surgical techniques, and reduce morbidity. Minimally invasive anterior antero-lateral approaches can substantially reduce the surgical morbidity of the approach and offer a better visualization of hidden areas like the contralateral vertebral body [45,46]. Navigation has become an indispensable tool to perform osteotomies at the right distance from the tumor in complex regions like the pelvis and spine [47,48]. Robotic-assisted steps during enbloc resection in the spine have already been reported [49]. The thread wire saw is the most frequently used tool to perform osteotomy or diskectomy, even if many surgeons prefer the bone scalpel, or the more current ultrasound scalpel [50]. A bone scalpel was used by Roy Camille to conclude osteotomy, reducing the risk of to damage the thecal sac by the uncontrolled use of a thread wire saw [6]. Protection of the thecal sac has been proposed by Tomita with manually managed special tools [7]. Recently, a safer device to protect the cord—connected to the rod—has been designed [51]. The thin thread wire saw remains today the more versatile tool and has been proposed as the key device of newly proposed techniques [52]. Also, in the case presented here, the thread wire has a very important role in an accurate spine osteotomy. In the case presented here, some peculiarities suggest surgical planning considerations not considered in the previously published classification [53]:

Location in the thoraco-lumbar region;

Huge anterior tumor extension (layer A) in close contact with the aorta and the cava;

Posterior part of the vertebral body (layer C) not involved by the tumor;

The tumor extended layers 5 to 8, fully saving the pedicles (sectors 4 and 9).

During the analysis of the imaging for the surgical planning, the possibility to save the canal circumference became evident, without obviously violating the tumor mass. There are two obvious consequences: significant reduction in the risk of neurological complications, and higher post-operative stability. However, the step to be solved was how to guide the thread wire saw along a non-linear path. The osteotomy had to run first on the coronal plane at an adequate distance from the posterior tumor margin for an oncologically appropriate resection, changing direction on the left side as the tumor expanded in the sub-pleural area at sector 4. A variant of the type 3 enbloc resection osteotomy [53] had to be planned to adapt the osteotomy line to the tumor extension and to save the canal circumference. The technical solution was to rely on navigation and robotic guidance to grant accuracy in the osteotomy lines with a few millimeters of tolerance, namely in the change in direction from the coronal plane to a sagittal plane. On the coronal plane, two K-wires, implanted using robotic assistance, guided the thread wire on the right coronal cut and a screw, positioned in the left pedicle of T9, acted as a pivot by which the saw could change direction to a sagittal plane to complete an “L-shaped” osteotomy. An anterior approach was decided as the first step to release the tumor mass from the aorta, aiming to leave a tumor-free margin. The sacrifice of the vessel and grafting had to be considered [17] if the aortic wall were to be found infiltrated or if the tumor were to be found completely surrounding the aorta. To this purpose, the vascular team participating in the operation were ready to perform a graft. Reconstruction of the continuity of the posterior vertebral wall and of the canal circumference allowed them to reduce the usual concern for the validity of the anterior support. A vascularized fibula was positioned abutting the preserved portion of the posterior wall, between the inferior endplate of T7 and the superior endplate of T11. A posterior construct was completed for the fixation T5 to T12. In the T8 and T10 pedicles, additional screws were implanted on the left side after tumor removal. Being manufactured in PRCF, it should not interfere with accelerated particle radiotherapy in case of local recurrence treatment.

## 4. Conclusions

This paper is intended to present an original technical solution to the performance of enbloc resection in a chordoma of the thoracic spine, unusually sparing the posterior part of the vertebral body, an area where most chordomas originate as best corresponding to the residual of notochord. The target of this technique is to spare the posterior wall and therefore the circumference of the spinal canal for the purpose of reducing the risk of neurological damage and to grant sounder stability. A marginal margin can be considered an acceptable result, but obviously the oncological outcome will be evaluated only in 5 years, as most local recurrence occurs later. During the anterior approach, the margin was achieved under direct visual control. All of the team were ready to perform a resection of the aorta and by-pass grafting in case of tumor involvement. This was not necessary, as a perimetral reactive tissue around the tumor, easily released from the aortic wall, was considered a safe margin. Navigation and robot-guided techniques made the “L” osteotomy possible, with a coronal leg performed at the best distance between the posterior tumor margin and the posterior vertebral wall and the shorter sagittal leg directed posteriorly into the pedicle at a distance from the costovertebral joint involved by the retropleural tumor extension. The key achievement was the idea to use a screw as a pivot to change the direction of the thread wire saw from the coronal to the sagittal plane.

## Data Availability

Research data are stored in the digital repository of Istituto Ortopedico Rizzoli.

## References

[1] Enneking W.F., Spanier S.S., Goodman M.A. (1980). A system for the surgical staging of musculoskeletal neoplasms. Clin. Orthop. Relat. Res..

[2] Simon M.A. (1988). Current concepts review: Limb salvage for Osteosarcoma. J. Bone Jt. Surg..

[3] Rosen G. (1985). Pre-operative (neo-adjuvant) chemotherapy for osteogenic sarcoma: A ten years experience. Orthopedics.

[4] Gitelis S., Mallin B.A., Piasecki P., Turner F. (1993). Intralesional excision compared with enbloc resection for giant cell tumor of bone. J. Bone Jt. Surg..

[5] Stener B., Johnsen O.E. (1971). Complete removal of three vertebrae for giant-cell tumour. J. Bone Jt. Surg. Br..

[6] Roy-Camille R., Montpierre H., Mazel C., Saillant G., Roy-Camille R. (1990). Technique de vertebrectomie totale lombaire. Rachis Dorsal et Lombaire Septieme Journees d’Orthopedie de la Pitié.

[7] Tomita K., Kawahara N., Baba H., Tsuchiya H., Fujita T., Toribatake Y. (2017). Total en bloc spondylectomy. A new surgical technique for primary malignant vertebral tumors. Spine.

[8] Fujita T., Kawahara N., Matsumoto T., Tomita K. (1999). Chordoma in the cervical spine managed with en bloc excision. Spine.

[9] Marmor E., Rhines L.D., Weinberg J.S., Gokaslan Z.L. (2001). Total en bloc lumbar spondylectomy. Case report. J. Neurosurg..

[10] Rhines L.D., Fourney D.R., Siadati A., Suk I., Gokaslan Z.L. (2005). En bloc resection of multilevel cervical chordoma with C-2 involvement. Case report and description of operative technique. J. Neurosurg. Spine.

[11] Currier B.L., Papagelopoulos P.J., Krauss W.E., Unni K.K., Yaszemski M.J. (2007). Total en bloc spondylectomy of C5 vertebra for chordoma. Spine.

[12] Amendola L., Cappuccio M., De Iure F., Bandiera S., Gasbarrini A., Boriani S. (2014). En bloc resections for primary spinal tumors in 20 years of experience: Effectiveness and safety. Spine J..

[13] Wang T., Jia Q., Fan R., Qian M., Yang C., Wei H., Liu T., Yang X., Wu Z., Zhao J. (2020). Multi-level En Bloc Resection as a Preferred Salvage Therapy for Recurrent Thoracolumbar Chondrosarcoma: A Comparative Study with Piecemeal Resection. Spine.

[14] Keynan O., Fisher C.G., Boyd M.C., O’Connell J.X., Dvorak M.F. (2005). Ligation and partial excision of the cauda equina as part of a wide resection of vertebral osteosarcoma: A case report and description of surgical technique. Spine.

[15] Murakami H., Tomita K., Kawahara N., Oda M., Yahata T., Yamaguchi T. (2006). Complete segmental resection of the spine, including the spinal cord, for telangiectatic osteosarcoma: A report of 2 cases. Spine.

[16] Krepler P., Windhager R., Toma C.D., Kitz K., Kotz R. (2003). Dura resection in combination with en bloc spondylectomy for primary malignant tumors of the spine. Spine.

[17] Gösling T., Pichlmaier M.A., Länger F., Krettek C., Hüfner T. (2013). Two-stage multilevel en bloc spondylectomy with resection and replacement of the aorta. Eur. Spine J..

[18] Somasundaram A., Wicks R.T., Lata A.L., Qasem S.A., Hsu W. (2015). En bloc spondylectomy for primary malignant fibrous histiocytoma of the thoracic spine with aortic involvement: Case report. J. Neurosurg. Spine.

[19] Avila M.J., Skoch J., Fennell V.S., Palejwala S.K., Walter C.M., Kim S., Baaj A.A. (2016). Combined posterior hemiosteotomies and stabilization with lateral thoracotomy for en bloc resection of thoracic paraspinal primary bone tumors: Technical note. J. Neurosurg. Spine.

[20] Huang W., Wei H., Cai W., Xu W., Yang X., Liu T., Wu Z., Huang Q., Yan W., Xiao J. (2018). Total En Bloc Spondylectomy for Solitary Metastatic Tumors of the Fourth Lumbar Spine in a Posterior-Only Approach. World Neurosurg..

[21] Yang X., Yang J., Jia Q., Zhong N., Jiao J., Hu J., Peng D., Liu W., Wan W., Xiao J. (2019). A Novel Technique for Total En bloc Spondylectomy of the Fifth Lumbar Tumor Through Posterior-Only Approach. Spine.

[22] Li Z., Lv Z., Li J. (2019). Total En Bloc Spondylectomy for the Fifth Lumbar Solitary Metastasis by a Posterior-Only Approach. World Neurosurg..

[23] Lu M., Zhou Z., Lei Z., Li H., Boriani S. (2019). Huge myxoid chondrosarcoma expanded into the thoracic cavity with spinal involvement. Eur. Spine J..

[24] Tang X., Cai Z., Wang R., Ji T., Guo W. (2021). En bloc resection and reconstruction of a huge chondrosarcoma involving multilevel upper thoracic spine and chest wall: Case report. BMC Musculoskelet. Disord..

[25] Ono K., Otsuki B., Fujibayashi S., Shimizu T., Murata K., Matsuda S. (2021). Subtotal En Bloc Resection of the Fourth Lumbar Vertebra for Giant Cell Tumor Using Combined Posterior and Lateral Retroperitoneal Approach. Spine Surg. Relat. Res..

[26] Aibara N., Watanabe K., Asano N., Suzuki S., Takahashi Y., Nori S., Tsuji O., Nagoshi N., Yagi M., Nakamura M. (2022). Bilateral Osteotomy of Pedicles for En Bloc Resection of a Malignant Tumor of the Posterior Thoracic Spine: A Case Report. JBJS Case Connect..

[27] Hasegawa K., Homma T., Hirano T., Ogose A., Hotta T., Yajiri Y., Nagano J., Inoue Y. (2007). Margin-free spondylectomy for extended malignant spine tumors: Surgical technique and outcome of 13 cases. Spine.

[28] Luzzati A.D., Shah S.P., Gagliano F.S., Perrucchini G.G., Fontanella W., Alloisio M. (2014). Four- and five-level en bloc spondylectomy for malignant spinal tumors. Spine.

[29] Mazel C., Owona P., Cogan A., Balabaud L., Grunenwald D. (2014). Long-term quality of life after en-bloc vertebrectomy: 25 patients followed up for 9years. Orthop. Traumatol. Surg. Res..

[30] Luzzati A.D., Shah S., Gagliano F., Perrucchini G., Scotto G., Alloisio M. (2015). Multilevel en bloc spondylectomy for tumors of the thoracic and lumbar spine is challenging but rewarding. Clin. Orthop. Relat. Res..

[31] Sciubba D.M., Ramos R.D.l.G., Goodwin C.R., Xu R., Bydon A., Witham T.F., Gokaslan Z.L., Wolinsky J.-P. (2016). Total en bloc spondylectomy for locally aggressive and primary malignant tumors of the lumbar spine. Eur. Spine J..

[32] Boriani S., Gasbarrini A., Bandiera S., Ghermandi R., Lador R. (2017). En Bloc Resections in the Spine: The Experience of 220 Patients During 25 Years. World Neurosurg..

[33] Zhang X.M., Fournel L., Lupo A., Canny E., Bobbio A., Lasry S., Regnard J.F., Sailhan F., Alifano M. (2019). En Bloc Resection of Thoracic Tumors Invading the Spine: A Single-Center Experience. Ann. Thorac. Surg..

[34] Dang L., Liu Z., Liu X., Jiang L., Yu M., Wu F., Wei F. (2020). Sagittal en bloc resection of primary tumors in the thoracic and lumbar spine: Feasibility, safety and outcome. Sci. Rep..

[35] Lu M., Zhou Z., Chen W., Lei Z., Dai S., Hou C., Du S., Jin Q., Jin D., Boriani S. (2022). En bloc resection of huge primary tumors with epidural involvement in the mobile spine using the “rotation-reversion” technique: Feasibility, safety, and clinical outcome of 11 cases. Front. Oncol..

[36] Yamazaki T., McLoughlin G.S., Patel S., Rhines L.D., Fourney D.R. (2009). Feasibility and safety of en bloc resection for primary spine tumors: A systematic review by the Spine Oncology Study Group. Spine.

[37] Tomita K., Kawahara N., Baba H., Tsuchiya H., Nagata S., Toribatake Y. (1994). Total en bloc spondylectomy for solitary spinal metastases. Int. Orthop..

[38] Boriani S. (2018). En bloc resection in the spine: A procedure of surgical oncology. J. Spine Surg..

[39] Boriani S., Weinstein J.N., Biagini R. (1997). Primary bone tumors of the spine. Terminology and surgical staging. Spine.

[40] Uri O., Folman Y., Laufer G., Behrbalk E. (2020). A Novel Spine Fixation System Made Entirely of Carbon-Fiber-Reinforced PEEK Composite: An In Vitro Mechanical Evaluation. Adv. Orthop..

[41] Ringel F., Ryang Y.M., Kirschke J.S., Müller B.S., Wilkens J.J., Brodard J., Combs S.E., Meyer B. (2017). Radiolucent Carbon Fiber-Reinforced Pedicle Screws for Treatment of Spinal Tumors: Advantages for Radiation Planning and Follow-Up Imaging. World Neurosurg..

[42] Ni X.-Y., Tang X.-B., Geng C.-R., Chen D. (2012). The prospect of carbon fiber implants in radiotherapy. J. Appl. Clin. Med. Phys..

[43] Mastella E., Molinelli S., Magro G., Mirandola A., Russo S., Vai A., Mairani A., Choi K., Fiore M., Fossati P. (2017). Dosimetric characterization of carbon fiber stabilization devices for post-operative particle therapy. Phys. Med..

[44] Pertsch N.J., Leary O.P., Camara-Quintana J.Q., Liu D.D., Niu T., Woo A.S., Ng T.T., Oyelese A.A., Fridley J.S., Gokaslan Z.L. (2021). A modern multidisciplinary approach to a large cervicothoracic chordoma using staged en bloc resection with intraoperative image-guided navigation and 3D-printed modeling: Illustrative case. J. Neurosurg. Case Lessons.

[45] Berjano P., Baroncini A., Cecchinato R., Langella F., Boriani S. (2023). En-bloc resection of a chordoma in L3 by a combined open posterior and less invasive retroperitoneal approach: Technical description and case report. Arch. Orthop. Trauma. Surg..

[46] Hireche K., Moqaddam M., Lonjon N., Marty-Ane C., Solovei L., Ozdemir B.A., Canaud L., Alric P. (2022). Combined video-assisted thoracoscopy surgery and posterior midline incision for en bloc resection of non-small-cell lung cancer invading the spine. Interact. Cardiovasc. Thorac. Surg..

[47] Ziu M., Traylor J.I., Paxman J., Gorrebeeck A., Fortes D.L. (2018). Utilizing Stereotactic Spine Navigation for Posterior Partial Vertebrectomy in an En Bloc Resection of a Superior Pulmonary Sulcus Tumor Invading the Thoracic Vertebrae: A Technical Note. Cureus.

[48] Tigchelaar S.S., Medress Z.A., Quon J., Dang P., Barbery D., Bobrow A., Kin C., Louis R., Desai A. (2022). Augmented Reality Neuronavigation for En Bloc Resection of Spinal Column Lesions. World Neurosurg..

[49] Corrado G., Zoccali C., Salducca N., Oddi A., Vizza E., Biagini R. (2019). Anterior robotic approach in en-bloc sacrectomy: A preliminary experience. J. Robot. Surg..

[50] Vedantam A., Vigneswaran K., Rao G., Walsh G.L., Rhines L.D., Tatsui C.E. (2020). Use of Navigated Ultrasonic Bone Cutting Tool for En Bloc Resection of Thoracic Chondrosarcoma: Technical Report. Oper. Neurosurg..

[51] Gasbarrini A., Simoes C.E., Amendola L., Bandiera S., Bròdano G.B., Cappuccio M., Boriani S. (2012). Influence of a thread wire saw guide and spinal cord protector device in “en bloc” vertebrectomies. J. Spinal Disord. Tech..

[52] Shah A.A., Pereira N.R.P., Pedlow F.X., Wain J.C., Yoon S.S., Hornicek F.J., Schwab J.H. (2017). Modified En Bloc Spondylectomy for Tumors of the Thoracic and Lumbar Spine: Surgical Technique and Outcomes. J. Bone Joint Surg. Am..

[53] Boriani S., Gasbarrini A., Ghermandi R., Fraifeld S., Cohen J.E., Kaplan L., Lenke L.G. (2023). Itshayek: Surgical Planning for EnBloc Resection of Spinal Tumors: Tailoring the Osteotomy to Tumor Extension. Corrective Osteotomies for Rigid Spinal Deformities.

